# Reliability and validity of physical examination tests for the assessment of ankle instability

**DOI:** 10.1186/s12998-022-00470-0

**Published:** 2022-12-19

**Authors:** Amber Beynon, Sylvie Le May, Jean Theroux

**Affiliations:** 1 Department of Chiropractic, Faculty of Medicine, Health and Human Sciences, Macquarie University, 75 Talavera Rd, Level 2, Sydney, NSW 2109 Australia; 2 Faculty of Nursing, University of Montreal, Montreal, 2900, Boul. Édouard-Montpetit, Montreal, QC H3T 1J4 Canada; 3 CHU Sainte-Justine Research Centre, 3175 Chemin de la Côte-Sainte-Catherine, Montreal, QC H3T 1C5 Canada; 4 College of Science, Health, Engineering and Education, Murdoch University, 90 South Street, Murdoch, WA 6150 Australia

**Keywords:** Ankle, Sprain, Reliability, Validity, Orthopaedic tests

## Abstract

**Introduction:**

Clinicians rely on certain physical examination tests to diagnose and potentially grade ankle sprains and ankle instability. Diagnostic error and inaccurate prognosis may have important repercussions for clinical decision-making and patient outcomes. Therefore, it is important to recognize the diagnostic value of orthopaedic tests through understanding the reliability and validity of these tests.

**Objective:**

To systematically review and report evidence on the reliability and validity of orthopaedic tests for the diagnosis of ankle sprains and instability.

**Methods:**

PubMed, CINAHL, Scopus, and Cochrane databases were searched from inception to December 2021. In addition, the reference list of included studies, located systematic reviews, and orthopaedic textbooks were searched. All articles reporting reliability or validity of physical examination or orthopaedic tests to diagnose ankle instability or sprains were included. Methodological quality of the reliability and the validity studies was assessed with The Quality Appraisal for Reliability studies checklist and the Quality Assessment of Diagnostic Accuracy Studies-2 respectively. We identified the number of times the orthopaedic test was investigated and the validity and/or reliability of each test.

**Results:**

Overall, sixteen studies were included. Three studies assessed reliability, eight assessed validity, and five evaluated both. Overall, fifteen tests were evaluated, none demonstrated robust reliability and validity scores. The anterolateral talar palpation test reported the highest diagnostic accuracy. Further, the anterior drawer test, the anterolateral talar palpation, the reverse anterior lateral drawer test, and palpation of the anterior talofibular ligament reported the highest sensitivity. The highest specificity was attributed to the anterior drawer test, the anterolateral drawer test, the reverse anterior lateral drawer test, tenderness on palpation of the proximal fibular, and the squeeze test.

**Conclusion:**

Overall, the diagnostic accuracy, reliability, and validity of physical examination tests for the assessment of ankle instability were limited. Physical examination tests should not be used in isolation, but rather in combination with the clinical history to diagnose an ankle sprain. Preliminary evidence suggests that the overall validity of physical examination for the ankle may be better if conducted five days after the injury rather than within 48 h of injury.

**Supplementary Information:**

The online version contains supplementary material available at 10.1186/s12998-022-00470-0.

## Introduction

Sprains have been found to be the most common type of ankle injuries [1, 2]. Persistent symptoms after ankle sprains are common [3–5]. Approximately 55% of individuals do not seek treatment for an ankle sprain [6]. and even when treatment is sought, treatment strategies are often insufficient in the rehabilitation and prevention of recurrences [7]. Consequently, ankle sprains may be underreported in certain populations, such as by athletes [7]. The first step in being able to improve patient outcomes for ankle sprains would be to correctly diagnose the ankle sprains. Clinicians rely on certain physical examination tests to diagnose and potentially grade ankle sprains and ankle instability. Diagnostic error and inaccurate prognosis may have important repercussions for clinical decision-making and patient outcomes [8]. Therefore, it is important to recognize the diagnostic value of orthopaedic tests through understanding the reliability and validity of these tests.

Reliability looks at the consistency demonstrated when a measure using a test is repeated [9]. Inter-rater reliability measures the reliability between two or more raters, and intra-rater reliability measures the reliability of the same rater on the same patient. Validity is the degree to which a test measures what it is intended to measure [9]. Determining the reliability and validity of a test or an examination technique is essential and provides credibility to the results obtained with the test or examination technique [10].

Several previous reviews have considered the diagnostic accuracy of particular ankle injuries. Schwieterman et al. [11] focussed their review on the ankle and foot special tests, including ligament stability, neurological issues, and tendons dysfunction. Schneiders et al. [12] and Netterström-Wedin et al. [13] specifically reviewed the diagnostic accuracy of clinical tests for low ankle sprain and included the drawer and talar tilt tests, while Sman et al. [14] assessed the accuracy of syndesmosis injuries specifically the squeeze test and the dorsiflexion-external rotation stress test. Finally, Delahunt et al. [15] published a consensus statement and recommendations focussing on developing a structured clinical assessment of acute lateral ankle sprain. This Delphi study included experts from the “International Ankle Consortium” executive committee [15]. Key recommendations included establishing the mechanism of injury and assessing ankle joint bones and ligaments. This group also established an “International Ankle Consortium Rehabilitation-Orientation Assessment (ROAST), hoping to help clinicians identify mechanical and sensorimotor impairments often found with chronic ankle instability [15]. They advocated that lateral ankle integrity, including syndesmosis, must be assessed, reporting that the most utilised clinical tests were the anterior drawer, talar tilt tests, syndesmosis direct palpation, and the squeeze test [15]. However, many primary studies do not clearly define or distinguish between the types of ankle sprains and often only consider the overall ankle injuries or ankle instability [16–19]. Therefore, focusing on one only component or considering only one type of ankle sprain in isolation may mean studies are missed.

Our objective was to systematically review and report evidence on the reliability and validity of physical examination (orthopaedic) tests for the diagnosis of ankle sprains and/or ankle instability.

## Methods

This review was prospectively registered within Prospero (CRD42019124090). This systematic review adheres to the Preferred Reporting Items for Systematic reviews and Meta-Analysis of Diagnostic Test Accuracy Studies (PRISMA-DTA) guidelines [20].

### Eligibility criteria

Studies regarding either the reliability or validity of manual physical examination or orthopaedic tests for the diagnosis of ankle instability or ankle sprains, including but not limited to anterior drawer test, talar tilt test, and external rotation test were included. We included original peer-reviewed studies written in English or French that included human participants of any age, gender, or ethnicity. Studies assessing validity had to include relevant statistical values such as odds ratios, predictive value, likelihood ratios, receiver operator curves, sensitivity, or specificity. Studies assessing reliability had to include relevant statistical values such as Kappa, intra-class correlation coefficient, or percent agreement.

### Search strategies

Searches were conducted in PubMed, CINAHL, Scopus, and Cochrane Database from inception to December 2021. In addition, reference lists of included studies, located systematic reviews, and important textbooks on orthopaedic evaluation/musculoskeletal diagnosis were searched for other possible studies [21–23].

**Fig. 1:**
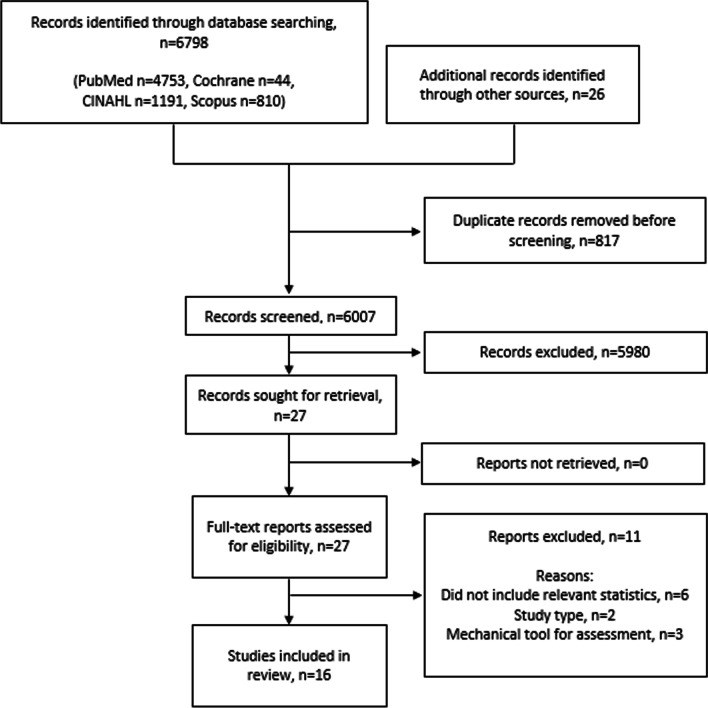
Study flow diagram

The keywords used combination were; “reproducibility of results”, “sensitivity and specificity”, joint instability, ligament, ankle, ankle joint, physical examination, validity, predictive value, accuracy, instability, laxity, injury, alignment, clinical assessment, palpation, orthopaedic, anterior drawer test, talar tilt, and external rotation test. The full search strategy for each database is included in Additional file 1. Search results were imported into bibliographic management software (EndNote X9.2) and duplicates discarded. Results of the search were reported as per the PRISMA flow diagram (See Fig. 1).

### Study selection and data extraction

Titles and abstracts were screened independently by two review authors (A.B and J.T) according to the eligibility criteria. The full texts of possibly relevant papers were obtained and again screened against the same criteria (A.B and J.T). Any disagreements were resolved through discussions and consensus between the reviewers.

Data from included studies were extracted independently by two reviewers (A.B and J.T), using data collection forms based on a Quality Appraisal for Reliability studies (QAREL) checklist [24] (reliability studies) and a Standards for Reporting Diagnostic Accuracy Studies (STARD) [25] (validity studies) by two review authors, and then collated together. Any disagreements were resolved through discussions and consensus between the reviewers. We extracted study characteristics, including purpose of study, sample size, study population, examiners, orthopaedic tests used, reference standards, and study results.

### Methodological quality assessment

The quality of included articles was assessed by two review authors. Methodological quality of the reliability studies was assessed with the QAREL checklist [24], which has 11 items covering seven domains including spectrum of subjects, spectrum of raters, rater blinding, order of examinations, suitable time intervals among repeated measures, test applied and interpreted correctly, and appropriate statistical analysis. Each item is rated as ‘Yes’, ‘No’, ‘Unclear’, or ‘Not applicable’. An item rated as ‘Yes’ indicates a good quality aspect of the study, while an item rated as ‘No’ indicates a poor quality assessment [24]. As recommended each quality item on the QAREL is considered separately rather than given an overall numerical quality score [24, 26]. Studies that were rated as ‘Yes’ on all items have an overall judgement of ‘high quality’. However, if a study is rated as ‘No’ or ‘Unclear’ on one or more items then it has an overall rating of ‘At risk of bias’.

Methodological quality of the validity of the studies was assessed using the Quality Assessment of Diagnostic Accuracy Studies 2 (QUADAS-2) [27]. The QUADAS-2 consists of four key domains covering patient selection, index test, reference standard, flow and timing, with each domain assessing risk of bias and three of the domains are also assessing applicability. As recommended, each domain on the QUADAS-2 is considered separately rather than giving an overall numerical quality score [24, 26, 27]. Studies that were rated as low risk on all domains regarding risk of bias or applicability have an overall judgement of ‘low risk of bias’ or ‘low concern regarding applicability’. However, if a study is rated as ‘high’ or ‘unclear’ in one or more domains then it has an overall evaluation of ‘at risk of bias’ or ‘concerns regarding applicability’ [27].

### Summary of findings

The characteristics of the included studies were tabulated for comparison. Identifying the number of times the orthopaedic test was investigated and the validity and/or reliability of each test. Where possible and appropriate (if studies included appropriate statistics), we have included a summary of the validity results summarised by test. Where possible further validity results were calculated from results provided within the included studies. Likelihood ratios were calculated if sensitivity and specificity were reported using the equations; positive likelihood ratio = sensitivity/(1-specificity) and negative likelihood ratio = (1-specificity)/sensitivity [9]. Predictive values and diagnostic accuracy were calculated if the true positive and negative, and false positive and negative values were reported [9]. The interpretation of Kappa values were based on the Landis and Koch reliability classification scale; below chance agreement < 0.00, slight agreement 0.00–0.20, fair agreement 0.21–0.40, moderate agreement 0.41–0.60, substantial agreement 0.61–0.80, and almost perfect agreement 0.81–1.00 [28]. Intra-class correlation coefficient (ICC) were interpreted as poor < 0.40, good 0.40–0.75, and excellent if > 0.75 [29].

We assessed whether results could be included into meta-analysis. Studies were assessed for statistical heterogeneity using I^2^ [30, 31]. Although there is no agreement on I^2^ interpretation, we applied the following criteria: 0–40% represented low heterogeneity, 30–60% represented moderate heterogeneity, 50–90% represented substantial heterogeneity, and 75–100% represented considerable heterogeneity [30]. When considering whether a meta-analysis is potentially suitable, we considered both the I^2^ and the methodological/clinical heterogeneity such as population under study, interpretation of index tests, and reference standards used.

## Results

### Study selection

We identified 6798 articles through searching databases and 26 additional records through other sources. After duplications were removed, 6007 articles remained. The title and abstract screen reduced the potential number down to 27 for full-text review. Eleven articles were excluded at full text review [32–42]. After the full-text review, 16 articles met the eligibility criteria (N = 935 participants) and are included in this review. Figure 1 outlines the screening and selection process.

### Study characteristics

Of the 16 included studies, three studies assessed reliability [17, 19, 43], eight studies assessed validity [16, 18, 44–49], and five studies assessed both reliability and validity [50–54]. Two studies were cadaveric studies [46, 51]. The characteristics of all included studies are reported in Table 1.

**Table 1:** Characteristics of included studies

Study	Purpose	Sample size	Study population	Examiner/s	Test/s	Reference standard	Statistic
Alonso et al. [43]	Interrater reliability	53	Patients with ankle injuries presenting to private physiotherapy clinics Time since injury: mean 34.2 ± 125 days (range 0–889). Mix acute/chronic 38 males (71.7%), 15 females (28.3%) Age: mean 24.3 ± 8.5 yrs. (range 12–52)	9 physiotherapists 1–11 yr. experience (mean 5 yrs.) 2 tested each participant	Squeeze test External rotation test The palpation test Dorsiflexion compression test	NA	Kappa, percent agreement
Croy et al. [44]	Validity	66	Individuals with a history of lateral ankle sprains. Time since injury: mean 23.1 ± 30.8 months (range 0.03, 108). Mix acute/chronic 35 males (53%), 31 females (47%) Age: mean 22.7 ± 3.6 yrs	Physical therapist 13 yr. experience	Anterior drawer test	Ultrasound	Sensitivity, specificity, likelihood ratios
de César et al. [45]	Validity	56	Patients with ankle sprains Time since injury: mean 6.6 ± 2.3 days Age: mean 32 ± 13 yrs. (range 18–66)	Lead investigator on study	Ankle external rotation test Squeeze test	MRI	Sensitivity, specificity
De Simoni et al. [49]	Validity	30	Patients with ankle sprains Time since injury: mean 3 days (range 0–19) 15 males (50%), 15 females (50%) Age: mean 33 yrs. (range 19–65)		Click test* Suction sign* Tenderness on palpation: Anterior talo-fibular ligament Calcaneo-fibular ligament	MRI	chi-squared test
George et al. [48]	Validity	35	Patients with a history of lateral ankle sprains Time since injury: mean 3.6 ± 3.32 weeks (between 5 days and 12 weeks) 17 males (48.6%), 18 females (51.4%) Age: mean 21.97 ± 7.1 yrs. (range 12–39)	1 sport medicine physician, 1 experienced musculoskeletal consultant radiologist	Anterior drawer test Talar tilt test	Ultrasound	Sensitivity, specificity, likelihood ratios, *p* value
Gomes et al. [16]	Validity	24	10 asymptomatic 14 complaints of ankle instability. Time since injury: mean 18.3 months (range 5–48) 9 males (64.3%), 5 females (35.7%) Age: mean 28 yrs. (range 23–42)	2 resident physicians trained by senior orthopaedic surgeon	Anterolateral talar palpation Anterior drawer test	MRI (only for cases)	Sensitivity, specificity, predictive values, accuracy
Großterlinden et al. [50]	Interrater reliability and validity	96	Patients with acute ankle sprains 55 males (57%), 41 females (43%) Age: mean 32.6 ± 10.2 yrs. (range 18–59)	2 examiners: 1 senior, 1 resident	Tenderness on palpation: Anterior inferior tibiofibular ligament Proximal fibula Deltoid ligament Anterior talo-fibular ligament Calcaneo-fibular ligament Syndesmosis Squeeze test External rotation test Drawer test Cotton test Crossed-leg test	MRI	Weighted Kappa, percent agreement, sensitivity, specificity, predictive values
Hosseinian et al. [53]	Interrater reliability and validity	105	Patients with ankle injuries presenting to a hospital. 47 male (55.2%), 58 female (55.2%). Age: mean 32.95 ± 1.55 yrs. (range 16–60)	2 examiners: 1 senior orthopedic resident 1 orthopedic specialist	Anterior drawer test Inversion stress test Eversion stress test Squeeze test External rotation stress test	MRI	Sensitivity, specificity, positive predictive value, negative predictive value, Kappa, *p* value
Li et al. [52]	Interrater reliability and validity	36	36 patients (72 ankles) with suspected anterior talofibular ligament injury 38 ankles (from 31 participants) injured group, 34 ankles (from 29 participants) control group Injured group: 18 males (58%), 13 females (42%). Age: mean 30.4 ± 8.9 yrs Control group: 15 males (52%), 14 females (48%) Age: mean 30.4 ± 8.9 yrs	2 examiners: 1 junior examiner 1 senior examiner	Anterior drawer test Anterolateral drawer test Reverse anterolateral drawer test	Ultrasound	Sensitivity, specificity, false negative rate, false positive rate, accuracy, Kappa, *p* value
Parasher et al. [17]	Intrarater/ interrater reliability	20	12 with ankle sprains, 8 without ankle sprains Measured bilaterally: 40 ankles (total: 16 ankle sprains, 24 injury free) 5 males (25%), 15 females (75%) Age: range 20–30 yrs	2 testers	Anterior drawer: goniometer Distal fibular position: digital vernier caliper	NA	Intra-class correlation coefficient
Phisitkul et al. [46]	Validity	10	Cadaveric: below the knee specimens (4 pairs, 2 single) 2 intact ligaments, 5 cut anterior talofibular ligament, 3 cut anterior talofibular and calcaneofibular ligament 4 males (6 ankles), 2 females (4 ankles) Age: mean 50 yrs	2 examiners: 1 Ankle surgeon (anteriolateral draw test) 1 In-training fellow (anterior drawer test)	Anterolateral drawer test Anterior drawer test	Cut ligaments. Direct anatomical measurement	ROC curve, sensitivity, specificity
Rosen et al. [18]	Validity	88	39 chronic ankle instability, 17 ankle sprain copers, 32 healthy controls 43 males (48.9%), 45 females (51.1%) Age: range 18–35 yrs	1 rater	Talar test: manual and Ligmaster	History: ankle injuries & Cumberland Ankle Instability Tool	Sensitivity, specificity, diagnostic odds ratio
Sman et al. [47]	Validity	87	Acute ankle sprains. 38 ankle syndesmosis injury, 42 Lateral sprain, 4 midfoot sprain, 1 medial ankle sprain, 2 pain no sprain Time since injury: mean 2.5 ± 3.8 days 78% male Age: mean 24.6 ± 6.5 yrs	13 clinicians: sports clubs, sports medicine, physiotherapy practices. 1–35 yrs. experience (mean 12 yrs.)	Dorsiflexion-external rotation test Dorsiflexion lunge with compression Squeeze test Syndesmosis ligament palpation	MRI	Sensitivity, specificity, likelihood ratios, accuracy, odds ratios
Van Dijk et al. [54]	Interrater reliability and validity	160	Patients with acute injury (within 48 h) to the lateral ligaments 116 males (72.5%, 44 females (27.5%) Age: mean 27.3 years (range 18–40 years)	5 examiners 1 experienced orthopaedic surgeon, 4 inexperienced doctors	Anterior drawer test Tenderness on palpation: Anterior talo-fibular Ligament Calcaneo-fibular Ligament Syndesmosis Medial Talocrural joint Peroneal tendon Lateral malleolus Diffusely lateral Supination line	Arthrography	Kappa, sensitivity, specificity
Vaseenon et al. [51]	Intrarater/ interrater reliability and validity	9	Cadaveric: human ankle specimens (4 pairs, 1 single) 3 intact ligaments, 3 cut anterior talofibular ligament, 3 cut anterior talofibular and calcaneofibular ligament 2 males, 3 females Age: mean 55 yrs. (range 48–70)	8 testers: 4 athletic training students (mean experience: 2.25 yrs.), 4 senior orthopaedic trainees (mean experience 4.5 yrs.)	Anterolateral drawer test Anterior drawer test	Cut ligaments. Direct anatomical measurement	Intra-class correlation coefficient, ROC, sensitivity, specificity,
Wilkin et al. [19]	Interrater reliability	60	38 sprainers, 22 non-sprainers, 3 additional ankle injuries 9 males (15%), 51 female (85%) Age: range 17–50 yrs	5 raters: 4 experienced physiotherapists, 1 undergraduate student (compared 2 experienced & student)	Anterior drawer in supine Anterior drawer in Crook lying Talar tilt Inversion tilt	NA	Intra-class correlation coefficient

NA, Not applicable; MRI, Magnetic resonance imaging; ROC curve, Receiver operating characteristics curve

*Not enough results in study to calculate the required statistics for results tables

### Methodological quality

Quality assessment of included reliability studies using QAREL is presented in Table 2. Only one study rated ‘yes’ on all 11 item yielding an overall judgement of ‘high quality’ [19]. The other six studies that assessed reliability had at least one item rated as ‘no’ or ‘unclear’ giving an overall judgement of ‘at risk of bias’ [17, 43, 50–54]. Common sources of bias included not enough information regarding blinding of the raters to the findings of other raters [17, 50–53], to their own prior findings [17, 43], to other clinical information [17, 43, 50, 53, 54], and to additional cues [17, 43, 50, 52–54]. All included studies used appropriate statistical tests.

**Table 2:** Quality assessment of included reliability studies using QAREL

Study	1	2	3	4	5	6	7	8	9	10	11
Alonso et al. [43]	Y	Y	Y	U	NA	N	U	U	Y	Y	Y
Großterlinden et al. [50]	Y	Y	U	NA	Y	N	N	U	Y	U	Y
Hosseinian et al. [53]	Y	Y	U	NA	Y	U	U	U	Y	U	Y
Li et al. [52]	Y	U	U	NA	Y	Y	U	U	U	Y	Y
Parasher et al. [17]	Y	U	U	U	NA	U	U	Y	Y	Y	Y
Van Dijk et al. [54]	Y	Y	Y	NA	Y	U	U	N	Y	U	Y
Vaseenon et al. [51]	N	Y	U	Y	Y	Y	Y	Y	U	Y	Y
Wilkin et al. [19]	Y	Y	Y	Y	Y	Y	Y	Y	Y	Y	Y

Y = yes, N = no, U = unclear, N/A = not applicable

Item 1: Was the test evaluated in a sample of subjects who were representative of those to whom the authors intended the results to be applied?

Item 2: Was the test performed by raters who were representative of those to whom the authors intended the results to be applied?

Item 3: Were raters blinded to the findings of other raters during the study?

Item 4: Were raters blinded to their own prior findings of the test under evaluation?

Item 5: Were raters blinded to the results of the reference standard for the target disorder (or variable) being evaluated?

Item 6: Were raters blinded to clinical information that was not intended to be provided as part of the testing procedure or study design?

Item 7: Were raters blinded to additional cues that were not part of the test?

Item 8: Was the order of examination varied?

Item 9: Was the time interval between repeated measurements compatible with the stability (or theoretical stability) of the variable being measured?

Item 10: Was the test applied correctly and interpreted appropriately?

Item 11: Were appropriate statistical measures of agreement used?

Quality assessment of included validity studies using QUADAS-2 are presented in Table 3. Four studies assessing validity had an overall judgement of ‘low risk of bias’ [46–48, 51], and seven studies had an overall judgement of ‘low concern regarding applicability’ [16, 18, 44, 45, 47–49]. Only two studies rated as ‘low risk of bias’ and ‘low concern of applicability’ [47, 48]. the other eight studies had at least one domain within risk of bias and/or applicability with a rating of ‘high’ or ‘unclear’ [16, 18, 44–46, 49–54]. Common sources of bias included not enough information on how the sample was enrolled [16, 44, 45, 52], how the index test was interpreted such as if a pre-specified threshold was used [16, 50, 52–54], if the reference standard was interpreted without knowledge of the test [44] or if the reference standard was likely to correctly classify the condition [18], and only the cases receiving the reference standard [16]. The two cadaveric studies posed concerns regarding the applicability of patient selection and the use of the reference standard [46, 51] therefore, the results from these studies will be reported separately.

**Table 3:**
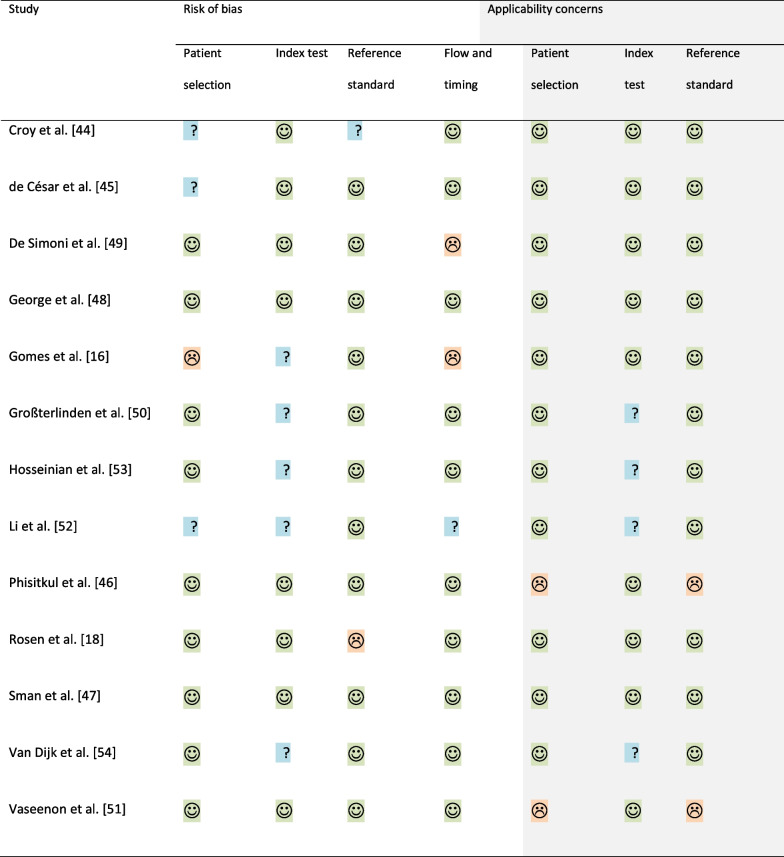
Quality assessment of included validity studies using QUADAS-2

Low Risk;  High Risk;  Unclear Risk

### Summary of findings

Six studies assessed the reliability of the anterior drawer test [17, 19, 50–53]. Three studies assessed the reliability of the external rotation test [43, 50, 53], and the squeeze test [43, 50, 53]. Two studies assessed the reliability of the anterolateral drawer test [51, 52], and the inversion tilt test [19, 53]. Only one study assessed the reliability of syndesmosis ligament palpation [43], the dorsiflexion compression test [43], tenderness of anterior inferior tibiofibular ligament, proximal fibular, deltoid ligament, anterior talofibular ligament and calcaneo-fibular ligament [50], the cotton test [50], the crossed-leg test [50], distal fibular position [17], the reverse anterolateral drawer test [52], talar tilt [19], and the eversion tilt test [53]. Table 4 reports an overview of the results from studies assessing reliability. Additional file 2 presents a description of all included tests based upon the provided reviewed literature.

**Table 4:** Results from studies assessing reliability

Study	Test	Intra-rater reliability	Inter-rater reliability
Alonso et al. [43]	Squeeze test	–	Kappa 0.50
	External rotation test	–	Kappa 0.75
	The palpation test (syndesmosis ligament)	–	Kappa 0.36
	Dorsiflexion compression test	–	Kappa 0.36
Großterlinden et al. [50]	*Tenderness on palpation*:		
	Anterior inferior tibiofibular ligament	–	Kappa 0.605
	Proximal fibula	–	Kappa 0.652
	Deltoid ligament	–	Kappa 0.646
	anterior talo-fibular ligament	–	Kappa 0.391
	Calcaneo-fibular ligament	–	Kappa 0.455
	Syndesmosis Squeeze test	–	Kappa 0.450
	External rotation test	–	Kappa 0.399
	Drawer test	–	Kappa 0.366
	Cotton test	–	Kappa 0.524
	Crossed-leg test	–	Kappa 0.440
	Overall rating	–	Kappa 0.626
Hosseinian et al. [53]	Anterior drawer test (anterior talofibular ligament)^a^	–	Kappa 0.356
	Anterior drawer test (anterior talofibular ligament)^b^	–	Kappa 0.461
	Anterior drawer test (anterior talofibular ligament)^c^	–	Kappa 0.349
	Inversion stress test (anterior talofibular ligament)^a^	–	Kappa − 0.093
	Inversion stress test (anterior talofibular ligament)^b^	–	Kappa 0.085
	Inversion stress test (anterior talofibular ligament)^c^	–	Kappa 0.214
	Inversion stress test (posterior talofibular ligament)^a^	–	Kappa 0.048
	Inversion stress test (posterior talofibular ligament)^b^	–	Kappa 0.025
	Inversion stress test (calcaneofibular ligament)^a^	–	Kappa 0.211
	Inversion stress test (calcaneofibular ligament)^b^	–	Kappa 0.399
	Inversion stress test (calcaneofibular ligament)^c^	–	Kappa 0.236
	Eversion stress test (deltoid ligament)^a^	–	Kappa 0.072
	Eversion stress test (deltoid ligament)^b^	–	Kappa 0.162
	Squeeze test (syndesmosis)^a^	–	Kappa 0.320
	Squeeze test (syndesmosis)^b^	–	Kappa 0.296
	External rotation stress test (syndesmosis)^a^	–	Kappa 0.255
	External rotation stress test (syndesmosis)^b^	–	Kappa 0.296
Li et al. [52]	Anterior Drawer test	–	Kappa 0.196
	Anterolateral drawer test	–	Kappa 0.528
	Reverse anterior drawer test	–	Kappa 0.639
Parasher et al. [17]	Anterior drawer: goniometer	Tester 1: ICC 0.96 (0.94, 0.98) Tester 2: ICC 0.97 (0.96, 0.98)	ICC 0.70 (0.48, 0.82)
	Distal fibular position: digital vernier caliper	Tester 1: ICC 0.97 (0.95, 0.98) Tester 2: ICC 0.91 (0.85, 0.95)	ICC 0.60 (0.40, 0.78)
Vaseenon et al. [51]	Anterolateral drawer test	ICC 0.8017	ICC 0.5230
	Anterior drawer test	ICC 0.9443	ICC 0.5274
Wilkin et al. [19]	Anterior drawer in supine	–	Experienced and student: ICC 0.16 (0.10, 0.33) Experienced raters: ICC 0.23 (− 0.02, 0.46)
	Anterior drawer in Crook lying	–	Experienced and student: ICC 0.06 (− 0.08, 0.23) Experienced raters: ICC − 0.12 (− 0.36, 0.14)
	Talar tilt	–	Experienced and student: ICC 0.33 (0.17,0.50) Experienced raters: ICC 0.22 (− 0.02, 0.45)
	Inversion Tilt	–	Experienced and student: ICC 0.29 (0.13, 0.46) Experienced raters: ICC 0.26 (0.00, 0.48)

ICC, Intra-class correlation coefficient

^a^Sprain + Partial tear + Complete tear

^b^Partial tear + Complete tear

^c^Complete tear

Nine studies assessed the validity of the anterior drawer test [16, 44, 46, 48, 50–54]. Four studies assessed the validity of the external rotation test [45, 47, 50, 53], and the squeeze test [45, 47, 50, 53]. Three studies assessed the validity of the anterolateral drawer test [46, 51, 52], and the tenderness of the anterior talofibular ligament and calcaneofibular ligament [49, 50, 54]. Two studies assessed the validity of a talar tilt test [18, 48], and tenderness of the syndesmosis [47, 54]. Only one study assessed the validity of dorsiflexion lunge with compression [47], tenderness of anterior inferior tibiofibular ligament [50], proximal fibular [50], deltoid ligament [50], medial ankle [54], talocrural joint [54], peroneal tendon [54], lateral malleolus [54], diffusely lateral [54], supination line [54], the cotton test [50], the crossed-leg test [50], the reverse anterolateral drawer test [52], the inversion stress test [53], and the eversion stress test [53]. Table 5 reports an overview of the results from studies assessing validity.

**Table 5:** Results from studies assessing validity

Study	Test	Sensitivity	Specificity	Positive LR	Negative LR	Positive PV	Negative PV	Accuracy
Croy et al. [44]	Anterior drawer test^a^	74 (58, 86)	38 (24, 56)	1.21 (0.86, 1.70)	0.66 (0.32, 1.36)	*57.8 (49.3, 65.4)*	*57.1 (39.4, 73.2)*	*57.6 (44.8, 69.7)*
	Anterior drawer test^b^	83 (64, 93)	40 (27, 56)	1.40 (1.03, 1.90)	0.41 (0.16, 1.08)	*36.4 (24.9, 49.1)*	*44.4 (37.1, 52.1)*	*56.1 (43.3, 68.3)*
	Anterior drawer test^c^	26 (14, 42)	67 (50, 81)	0.79 (0.37, 1.74)	1.09 (0.80, 1.49)	*47.4 (29.6, 65.8)*	*44.7 (37.2, 52.5)*	*45.5 (33.1, 58.2)*
	Anterior drawer test^c^	33 (18, 53)	73 (59, 85)	1.27 (0.59, 2.72)	0.90 (0.64, 1.26)	*36.4 (24.8, 49.1)*	*66.0 (58.1, 73.0)*	*59.1 (46.3, 71.1)*
de César et al. [45]	Ankle external rotation test	20	84.8	*1.32*	*0.94*			
	Squeeze test	30	93.5	*4.62*	*0.75*			
	Both tests with physical exam	40	84.8	*2.63*	*0.71*			
De Simoni et al. [49]	*Tenderness on palpation*:							
	Anterior talo-fibular ligament	*100 (88, 100)*				*100*		
	Calcaneo-fibular ligament	*68 (46, 85)*	*40 (5, 85)*	*1.13 (0.53, 2.43)*	*0.80 (0.24, 2.70)*	*85.0 (72.5, 92.4)*	*20.0 (6.9, 45.8)*	*63.3 (43.9, 80.1)*
George et al. [48]	Anterior drawer test	59 (36, 79)	100 (78, 100)	0.77	0.44	*100*	*59.1 (46.6, 70.5)*	*74.3 (56.7, 87.5)*
	Talar tilt test	54 (23, 83)	100 (85, 100)	1.00	0.45	*100*	*82.8 (71.5, 90.2)*	*85.7 (69.7, 95.2)*
Gomes et al. [16]	Anterior drawer test	50	100	*	*0.5*	100	56.3	69.6
	Anterolateral talar palpation	100	77.8	*4.5*	*0*	87.5	100	91.3
Großterlinden et al. [50]	*Tenderness on palpation*:							
	Anterior inferior tibiofibular	41.7	52.5	*0.88*	*1.11*	34.1	59.6	
	Proximal fibula	7.7	93.9	*1.26*	*0.98*	16.7	86.7	
	Deltoid ligament	33.3	69.5	*1.09*	*0.96*	38.7	63.1	
	Anterior talo-fibular ligament	77.8	27.1	*1.07*	*0.82*	38.9	66.7	
	Calcaneo-fibular ligament	61.1	47.5	*1.16*	*0.82*	41.5	67.4	
	Syndesmosis Squeeze test	44.4	55.9	*1.01*	*0.99*	37.2	62.3	
	External rotation test	55.6	47.5	*1.06*	*0.93*	38.5	63.6	
	Drawer test	44.4	67.8	*1.38*	*0.82*	44.4	66.7	
	Cotton test	30.6	67.8	*0.95*	*1.02*	35.5	61.5	
	Crossed-leg test	13.9	83.1	*0.82*	*1.04*	33.3	61.3	
Hosseinian et al. [53]	Anterior drawer test (anterior talofibular ligament)^e^	81	80	*4.05*	*0.24*	97	30.8	
	Anterior drawer test (anterior talofibular ligament)^f^	85	63	*2.30*	*0.24*	89	54	
	Anterior drawer test (anterior talofibular ligament)^g^	42	94	*7.00*	*0.62*	88	59	
	Inversion stress test (anterior talofibular ligament)^e^	30	40	*0.50*	*1.75*	84	4	
	Inversion stress test (anterior talofibular ligament)^f^	46	68	*1.44*	*0.79*	84	25	
	Inversion stress test (anterior talofibular ligament)^g^	67	54	*1.46*	*0.61*	62	60	
	Inversion stress test (posterior talofibular ligament)^e^	50	58	*1.19*	*0.86*	17	87	
	Inversion stress test (posterior talofibular ligament)^f^	100	58	*2.38*	*0*	2	100	
	Inversion stress test (calcaneofibular ligament)^e^	50	86	*3.57*	*0.58*	93	30	
	Inversion stress test (calcaneofibular ligament)^f^	65	75	*2.60*	*0.47*	73	66	
	Inversion stress test (calcaneofibular ligament)^g^	63	80	*3.15*	*0.46*	95	26	
	Eversion stress test (deltoid ligament)^e^	70	98	*35.00*	*0.31*	75	63	
	Eversion stress test (deltoid ligament)^f^	17	97	*5.67*	*0.86*	25	95	
	Squeeze test (syndesmosis)^e^	25	99	*25.00*	*0.76*	87	79	
	Squeeze test (syndesmosis)^f^	33	95	*6.60*	*0.71*	94	37	
	External rotation stress test (syndesmosis)^e^	22	97	*7.33*	*0.80*	75	78	
	External rotation stress test (syndesmosis)^f^	33	95	*6.6*	*0.71*	37	94	
Li et al. [52]	Anterior Drawer test	53^h^, 39.5^i^	100^h^, 100^i^	*	0.95^h^, 0.61^i^			50^h^, 68.1^i^
	Anterolateral drawer test	44.7^h^, 50^i^	100^h^, 97.1^i^	*^h^, 17.2^i^	0.55^h^, 0.51^i^			70.8^h^, 72.2^i^
	Reverse anterior drawer test	86.8^h^, 92.1^i^	91.2^h^, 88.2^i^	9.9^h^, 7.8^i^	0.14^h^, 0.09^i^			88.9^h^, 90.3^i^
Phisitkul et al. [46]	Anterolateral drawer	100	100	*	*0*			
	Anterior drawer test	75	50	*1.5*	*0.5*			
Rosen et al. [18]	Manual talar tilt test	49 (34, 64)	82 (69, 90)	2.65 (1.35, 5.20)	0.63 (0.45, 0.88)			
Sman et al. [47]	Dorsiflexion-external rotation test	71 (55, 83)	63 (49, 75)	1.93 (1.28, 2.94)	0.46 (0.27, 0.79)	*60.0 (49.6, 69.5)*	*73.8 (62.1, 82.9)*	66.7 *(55.7, 76.4)*
	Dorsiflexion lunge with compression	69 (53, 82)	41 (28, 56)	1.18 (0.86, 1.64)	0.74 (0.41, 1.35)	*48.1 (40.1, 56.2)*	*63.3 (48.6, 75.9)*	53.7 *(42.3, 64.7)*
	Squeeze test	26 (15, 42)	88 (76, 94)	2.15 (0.86, 5.39)	0.84 (0.68, 1.04)	*62.5 (39.9, 80.7)*	*60.6 (55.3, 65.6)*	60.9 *(49.9, 71.2)*
	Syndesmosis ligament palpation	92 (79, 97)	29 (18, 42)	1.29 (1.06, 1.58)	0.28 (0.09, 0.89)	*50.0 (45.0, 55.0)*	*82.3 (59.1, 93.8)*	56.3 *(45.3, 66.9)*
Van Dijk et al. [54]	Anterior drawer test	*80 (72, 87)*	*68 (50, 82)*	*2.48 (1.54, 3.98)*	*0.29 (0.19, 0.45)*	*88.7 (83.0, 92.6)*	*52.1 (41.4, 62.5)*	*77.3 (69.8, 83.6)*
	Tenderness on palpation							
	Anterior talo-fibular ligament	*100 (97, 100)*	*32 (18, 49)*	*1.46 (1.18, 1.81)*	*0*	*82.4 (79.1, 85.4)*	*100*	*83.8 (77.1, 89.1)*
	Calcaneo-fibular ligament	*49 (40, 58),*	*76 (60, 89)*	*2.08 (1.14, 3.78)*	*0.67 (0.52, 0.85)*	*87.0 (78.6, 92.4)*	*32.0 (26.7, 37.5)*	*55.6 (47.6, 63.5)*
	Syndesmosis	*43 (35, 53)*	*89 (75, 97)*	*4.13 (1.60, 10.66)*	*0.63 (0.52, 0.76)*	*93.0 (83.7, 97.2)*	*33.0 (28.9, 37.3)*	*54.4 (46.3, 62.3)*
	Medial	*52 (43, 62)*	*58 (41, 74)*	*1.25 (0.83, 1.88)*	*0.82 (0.59, 1.14)*	*80.0 (72.7, 85.8)*	*27.5 (21.4, 34.5)*	*53.8 (45.7, 61.7)*
	Talocrural joint	*23 (16, 31)*	*97 (86, 1.00)*	*8.72 (1.23, 61.99)*	*0.79 (0.71, 0.88)*	*96.6 (79.8, 99.5)*	*28.2 (26.1, 30.5)*	*40.6 (32.9, 48.7)*
	Peroneal tendon	*26 (19, 35)*	*92 (79, 98)*	*3.32 (1.08, 10.24)*	*0.80 (0.70, 0.92)*	*91.4 (77.6, 97.1)*	*28.0 (25.3, 30.9)*	*41.9 (34.1, 49.9)*
	Lateral malleolus	*16 (10, 23)*	*95 (82, 99)*	*2.96 (0.72, 12.13)*	*0.89 (0.80, 0.99)*	*90.5 (69.9, 97.5)*	*25.9 (23.9, 28.0)*	*34.4 (27.1, 42.3)*
	Diffusely lateral	*3 (1, 8)*	*100 (91, 100)*	*	*0.97 (0.94, 1.00)*	*100*	*24.4 (23.8, 25.0)*	*26.3 (19.6, 33.8)*
	Supination line	*3 (1, 8)*	*71 (54, 85)*	*0.11 (0.04, 0.34)*	*1.36 (1.11, 1.67)*	*26.7 (10.9, 51.8)*	*18.6 (15.7, 21.9)*	*19.4 (13.6, 26.4)*
Vaseenon et al. [51]	Anterolateral drawer test	100	66.67	*3*	*0*			
	Anterior drawer test	100	66.67	*3*	*0*			

LR: likelihood ratio, PV: predictive value

^*^Specificity is 100% therefore it is not possible to calculate + LR.. *Results in italics* indicate numbers that were calculated for this review from provided results in the study

^a^Grade 2 or above considered positive: 2.3 mm or greater

^b^Grade 2 or above considered positive: 3.7 mm or greater

^c^Grade 3 or above considered positive: 2.3 mm or greater

^d^Grade 3 or above considered positive: 3.7 mm or greater

^e^Sprain + Partial tear + Complete tear

^f^Partial tear + Complete tear

^g^Complete tear

^h^Junior examiner

^i^Senior examiner

Due to the methodological and statistical heterogeneity of the included studies, a meta-analysis was not possible. When combining results, the I^2^ value was 75–100% representing considerable heterogeneity for all considered meta-analyses. Additionally, there was major methodological and clinical heterogeneity among the included studies. For example, nine included studies assessed the validity of the anterior drawer test. However, two of these studies are cadaveric studies [46, 51]. A range of different reference standards were used within these studies, including ultrasound [44, 48, 52], MRI [16, 50, 53], arthrography [54], and cutting the ligaments and measured with direct anatomical measurements [46, 51]. There were also differences in how the anterior drawer test was conducted and scores interpreted.

There were only three tests; anterior drawer [17, 51], distal fibular position [17], and anterolateral drawer tests [51], that had results reported regarding intra-rater reliability. These tests were all reported to have excellent intra-rater reliability [17, 51]. However, these results are only based on at most two studies [17, 51], in which one of these studies was using cadavers [51]. The two tests with the highest reported inter-rater reliability were the external rotation and the anterior drawer tests, rated as substantial [43] and good [17] agreement respectively. However, other studies have rated the inter-rater reliability of the anterior drawer test as slight [52] and poor [19], and the external rotation test as fair [50, 53], demonstrating inconsistent results. The only test to show some consistent results based on more than one included study was the squeeze test, which was rated as having moderate inter-rater reliability based on results from two studies [43, 50].

Overall, the test with the highest reported diagnostic accuracy (91.3%) was the anterolateral talar palpation test, however, this was only based on the results of one study [16]. The tests with the highest reported sensitivity were the anterior drawer test [44, 51, 53], the anterolateral talar palpation [16], the reverse anterior lateral drawer test [52], and palpation of the anterior talofibular ligament [49, 54]. However, there were quite inconsistent results with lower sensitivity reported for the anterior drawer test depending on the grade of the ankle sprain to indicate positive test results [44]. The anterior drawer test also reported the lowest negative likelihood ratio (0.24) compared to other reported tests assessing validity for ankle sprains [53]. The tests with the highest reported specificity were the anterior drawer [16, 48, 52, 53], anterolateral drawer test [46, 52], the reverse anterior lateral drawer test [52], tenderness on palpation of the proximal fibular [50] and diffusely lateral [54], the squeeze test [45, 47, 53], the talar tilt test [48], and the eversion stress test [53]. Again, there were inconsistent results with lower specificity results reported for the anterior drawer test in other studies [44, 46, 50, 51]. The squeeze test reported the highest positive likelihood ratio (35) compared to all other reported tests [53]. The reverse anterolateral drawer test reported both a very high sensitivity and specificity, but this was only reported within one study [52].

### Consideration of type of ankle sprain

In the diagnosis of an ankle injury, the mechanism of injury should be considered, such as by using Lauge-Hansen classification [55]. While many included studies included a mixture of participants with different types of ankle sprains, some included studies did specify which tests should be used for which type of ankle injury. Orthopaedic tests to assess for a potential syndesmosis injury include; tenderness of palpation of direct ligaments [43, 47, 50], squeeze test [43, 47, 50], external rotation stress test [43, 50, 53], dorsiflexion compression test [43, 47], cotton test [50], and crossed-leg test [50]. Orthopaedic tests to assess for a potential lateral ligament injury include; anterior drawer test [44, 46, 51–53], anterolateral drawer test [46, 51, 52], anterolateral talar palpation, reverse anterolateral drawer test [52], tenderness of palpation of direct ligaments [50], inversion stress test [53], and talar tilt test. Orthopaedic tests to assess for a potential medial ligament injury include; tenderness of palpation of direct ligaments [50], and eversion stress test [53]. Additional file 3 reports orthopaedic tests for different types of ankle sprains. Additional file 4 reports a summary of the sensitivity and specificity values by orthopaedic test.

## Discussion

The tests reviewed included the anterior drawer, anterolateral drawer, reverse anterolateral drawer test, external rotation, dorsiflexion external rotation, squeeze, palpation and tenderness, cotton, crossed-leg, dorsiflexion compression, distal fibular position, talar tilt, inversion tilt, eversion stress, and dorsiflexion lunge with compression tests. Overall, none of these tests have shown robust reliability and validity scores. Even the studies that used a combination of tests did not show high diagnostic accuracy [47]. However, one study did find that the overall validity of physical examination for the ankle did drastically increase if conducted five days after the injury rather than within 48 h of injury [54]. The orthopaedic tests should be used in combination with the clinical history.

Many of the included studies had different or unclear definitions of ankle sprains. These could include a mixture of participants with a history of lateral, medial and/or syndesmotic ankle sprains [16–19, 49, 54]. Many studies had a mixture of acute and chronic ankle sprains [16, 17, 43, 44] or no information regarding how long the injury was ongoing [17, 19]. The clinical usefulness of certain tests could differ among acute or chronic conditions. Also, some studies did not consider the grade of the ankle sprain required to indicate a positive test [16, 17]. One study that did consider the grade of the ankle sprain showed that when a higher grade (grade 3 or above) was used to consider a positive result, they observed a higher specificity but a lower sensitivity compared to values when using a grade 2 or above [44].

There were other differences in how the studies were conducted, which hindered the interpretation of this systematic review’s results. There were a range of different reference tests used, including ultrasound [44, 48, 52], MRI [16, 45, 47, 49, 50, 53], Cumberland ankle instability tool [18], arthrography [54], and cutting the ligaments to directly measure anatomical movements [46, 51]. Additionally, there were differences in how tests were conducted, and scores interpreted. For instance, some authors used subjective or objective interpretations to assess the drawer test, such as feeling if there is any laxity [19, 44] compared to using a goniometer [17]. Other studies did not provide enough detail about how the index test was interpreted such as if a pre-specified threshold was used [16, 50, 52, 53]. Furthermore, many studies had a mixture of examiners with varying degrees of experience from students or clinicians with minimal clinical experience to highly experienced clinicians [19, 43, 46, 47, 50–52]. When studies compared the results between students or junior examiners compared to more senior or experienced examiners, there were mixed results. On occasions, the less experienced examiners yielded higher results and on other occasions, the more experienced examiners yielding higher results [19, 52]. Moreover, the two studies using cadaveric specimens [46, 51] posed concerns regarding the applicability to a clinical population, there would be differences between using living participants compared to using cadaveric specimens. The advantage of using cadaveric specimens over live patients is the easiness of distinguishing between a true positive or a true negative as the ligaments were cut however, it lacks important feedback such as patient cues and tenderness.

This systematic review differs from previous reviews. Two previous reviews on ankle injuries were published six [12] and nine [11] years ago. While both reviews investigated the diagnostic accuracy of special ankle tests, Schneiders et al. [12] included special tests of ankle and foot musculoskeletal pathologies, and Schneiders et al. [12] reviewed publications that included only the two most widely used clinical tests to assess lateral ankle sprains, namely the anterior drawer and the talar tilt tests. Both these review articles [11, 12] did not account for the reliability of the index tests. A more recent review [13] looked at the accuracy of clinical tests assessing ligamentous injury of the talocrural and subtalar joint. Netterström-Wedin et al. [13] focussed on lower lateral ankle stability assessment and did not review ankle stability integrity in its entirety, including the ankle medial side and higher aspect (syndesmosis), which we have considered in our systematic review. We also evaluated the reliability of those tests. Considering our review objectives, we included studies [17, 18, 43, 45–47, 50, 51, 53, 56] that were not included by Netterström-Wedin et al. [13].

Considering the risk of bias assessment of similar included studies to the most recent previous systematic review [13], our interpretation of the QUADAS-2 tool differed for some studies. For example, Netterström-Wedin et al. [13] reported that Li et al. [52] was at low risk of bias and low applicability concerns on all items. We considered this same article to have patient selection and index test to be rated as ‘unclear risk of bias, and ‘unclear’ concerns regarding the applicability of the index test, due to the study not including enough details. These bias assessment discrepancies probably relate to the subjective interpretation of the tool which has been reported with other measurement tools [57, 58] the agreement appears to be lowest on highly subjective items. Reliability may vary according to reviewers' familiarity with the tool, their expertise, items’ interpretation, or whether reviewers have worked together before [57]. What is important is to apply the risk of bias tool consistently within the systematic review. Considering this subjectivity, comparing similar systematic reviews becomes challenging.

Despite the concerns raised by our systematic review on the diagnostic value of the included ankle physical tests, clinicians should not dismiss the significance of a thorough physical examination. The argument supporting technology as a substitute remains notably debatable, often associated with false-positive results [59], imparting a false sense of confidence that can sometimes delay and increase the burden of care. Similar to Rheumatology which lacks a specific organ or system constraint [60], musculoskeletal complaints involve multiple tissues and remain a common reason for patients visiting their primary health practitioners [61]. Despite that, the physical examination, including its orthopaedic component, remains a neglected field of research [62], this component should not be abandoned but instead better understood and refined [63].

### Strengths and limitations of this review

This systematic review endeavoured to include all relevant articles that assessed the reliability and/or validity of any type of ankle sprain and/or ankle instability and included a wide initial search strategy. The methodological quality of all included studies was assessed by using the QAREL and/or the QUADAS-2. Due to the methodological heterogeneity of the included studies no meta-analysis could be conducted. The results from this review highlight the heterogeneity within the current literature. Additionally, results are only based on a few studies at most for each test, frequently with limited sample sizes. This systematic review was limited to studies written in English and French.

### Recommendations for future research

Appropriate reference standards should be used when determining the diagnostic accuracy of physical examination tests. More high-quality research is needed to truly determine the reliability and validity of physical examination tests for the diagnoses of ankle sprains. Clear definitions of the type of ankle injury and the duration of time since the injury should be considered in future research. Furthermore, to truly consider the use of physical examination tests in a clinical and pragmatic way, future studies should use a combination of clinical tests along with the patient’s history.

### Clinical implications

Although individual orthopaedic tests may not yield high reliability and validity, they should not be discarded entirely. When examining a patient with an ankle injury, fractures of the ankle and mid-foot should first be excluded, such as by using the Ottawa ankle rules [64], and then consider a range of orthopaedic tests to assess for an ankle sprain. Physical examination tests should not be used in isolation; instead, in combination with the clinical history to diagnose an ankle sprain. Careful consideration should be taken as to when is the most appropriate time to conduct the physical examination.

## Conclusion

The diagnostic accuracy, reliability, and validity of physical examination tests for the assessment of ankle instability were limited. Physical examination tests should not be used in isolation to diagnose an ankle sprain. Rather clinicians should use a combination of physical examination tests along with the clinical history. Future studies should ensure appropriate reference standards are used, such as MRI or arthroscopy, and use a combination of clinical tests with the patient’s history to determine the diagnostic accuracy in a clinical and pragmatic way.

## Supplementary Information

**Additional file 1**. Search strategy.

**Additional file 2**. Tests description.

**Additional file 3**. Orthopaedic tests for different types of ankle sprains.

**Additional file 4**. Summary of the sensitivity and specificity values by orthopaedic test.

## Data Availability

All data generated or analysed during this study are included in this published article and its supplementary information files.
