# Supplementary material for: Reliability and validity of physical examination tests for the assessment of ankle instability
Source: Chiropr Man Therap. 2022 Dec 19;30:58. doi: 10.1186/s12998-022-00470-0 (PMC9764698; doi:10.1186/s12998-022-00470-0)
Supplement: Supplementary file 1 — Additional file 1. Search strategy. [file 12998_2022_470_MOESM1_ESM.pdf]

Additional File 1. Search strategy

PUBMED
(((((reliab* OR reproduc* OR validity OR accuracy OR variability OR “predictive value”)) OR (interobserver* OR intertester* OR interrater* OR interexamin* OR intraobserver* OR intratester* OR intrarater* OR intraexamin* OR “observer variation” [Mesh])) OR ((inter OR intra) AND (rater* OR examin* OR tester* OR observer* OR “sensitivity and specificity” [MESH] OR “reproducibility of results”)))) AND ((((patholog* OR lesion* OR ruptur$ OR torn OR tear* OR trauma OR traumas OR effusion* OR instabilit* OR “joint instability” [Mesh] OR laxity OR injur* OR disorder* OR syndrome OR pain OR alignment)) AND ankle OR “ankle” [Mesh] OR “ankle joint” [Mesh] AND (“physical examination” OR “physical examination” [Mesh] OR “clinical assessment” OR test OR manual* OR manoeuv* OR maneuv* OR palpation OR examiner* OR orthopaedic OR “anterior drawer test” OR “talar tilt” OR “external rotation test”))

CINAHL

1. patholog* or lesion* or ruptur* or torn or tear* or trauma$ or effusion* or instabilit* or laxity or injur* or disorder* or syndrome or pain or alignment
2. ankle OR “ankle ligament” OR “ankle joint”
3. (“physical examination” OR “physical examination” [Mesh] OR “clinical assessment” OR test OR manual* OR manoeuv* OR maneuv* OR palpation OR examiner* OR orthopaedic OR “anterior drawer test” OR “talar tilt” OR “external rotation test”))
4. (((((reliab* OR reproduc* OR validity OR accuracy OR variability OR “predictive value”)) OR (interobserver* OR intertester* OR interrater* OR interexamin* OR intraobserver* OR intratester* OR intrarater* OR intraexamin* OR “observer variation”)) OR ((inter OR intra) AND (rater* OR examin* OR tester* OR observer* OR “sensitivity and specificity” OR “reproducibility of results”))))
5. S1 AND S2 AND S3 AND S4

Cochrane Library

1. Reliability OR validity OR reproducibility of results OR accuracy
2. joint instability OR instabilit* OR patholog* OR lesion* OR rupture OR torn OR tear*
3. ankle OR “ankle ligament” OR “ankle joint”
4. (“physical examination” OR “clinical assessment” OR test OR orthopaedic OR “anterior drawer test” OR “talar tilt” OR “external rotation test”)
5. S1 AND S2 AND S3 AND S4

Scopus
(Reliability OR validity) AND (joint instability OR instabilit* OR rupture OR torn OR tear) AND (ankle OR {ankle ligament} OR {ankle joint}) AND ({physical examination} OR {clinical assessment} OR orthopaedic)
