# Supplementary material for: Reliability and validity of physical examination tests for the assessment of ankle instability
Source: Chiropr Man Therap. 2022 Dec 19;30:58. doi: 10.1186/s12998-022-00470-0 (PMC9764698; doi:10.1186/s12998-022-00470-0)
Supplement: Supplementary file 2 — Additional file 2. Tests description. [file 12998_2022_470_MOESM2_ESM.pdf]

Additional File 2. Tests description

Anterior drawer test
The patient is seated with their calf hanging over the edge of the examination bed. The examiner stabilized the patient's distal tibia with one hand and applies an anteriorly directed force to the calcaneus with the other hand.
Anterior drawer in Crook lying
The patient is lying with their hip flexed and knee bent 90 degrees, the feet resting on the table (Crook lying). The examiner applies an anteroposterior force over the anterior aspect of the distal tibia and fibula moving the distal tibio-fibular articulation over the talus.

Anterolateral drawer test
The patient is seated with their calf hanging over the edge of the examination bed. The examiner stabilized the patient's distal tibia with one hand and provided a combination of an anteriorly oriented force, measurement of the talus displacement, and control the ankle plantarflexion (10 – 15^0) simultaneously with the other hand. The examiner's thumb is placed along the relatively smooth plane of the lateral aspect of the anterior talar dome and the anterior aspect of the lateral malleolus, 1cm proximal to its tip.

Reverse anterolateral drawer test
The patient is supine with their knee flexed at an angle that facilitate the ankle plantarflexion (10 – 15^0). The patient's heel is stabilised on the table not restraining the ankle internal rotation. The examiner's thumb is placed along the relatively smooth plane of the lateral aspect of the anterior talar dome and the anterior aspect of the lateral malleolus, 1cm proximal to its tip. A posterior force, parallel with to the articular surface arch of the talus is applied by the examiner over the distal tibia

External rotation test (Dorsiflexion-external rotation test)
The patient is sitting over the side of the table. The examiner brings the ankle into maximal dorsiflexion and applies a passive external rotation stress over the ankle and foot while the patient's knee is maintained at 90 degrees.

Squeeze test
The examiner applies a manual compression of the fibula to the tibia above the midpoint of the calf.

Dorsiflexion-compression test (dorsiflexion lunge with compression)
The patient is standing with their knee bent and ankle in dorsiflexion without rising the heel from the ground (the patient actively dorsiflexes his ankle). This position simulate active syndesmosis loading. The test is initially performed without the examiner assistance and again with examiner actively applying compression around the malleoli.

Crossed-leg test
The patient is seated and asked to cross the affected leg over the opposite knee. The clinician then applies pressure to the proximal fibula of the affected leg.

The Cotton Test
The examiner stabilises the distal tibia and fibula with one hand while applying a lateral translation force with the other hand. The test is said to be positive if a translation occurs that is greater the 3 – 5 mm or if a clunk is heard or felt.

Talar tilt test
The patient is sitting with the examined ankle freely hanging off the table. This test can be performed using three different ankle position, plantarflexion, anatomical position, or maximal dorsiflexion. These positions impart a greater stress over the anterior talofibular ligament, calceneo-fibular (lateral ankle) and deltoid (medial ankle) ligament and the posterior talofibular ligament respectively. The examiner stabilises the distal tibia and fibula with one and the calcaneus with the other hand. The examiner then brings the patient's ankle into inversion or eversion testing ankle lateral or medial laxity.
