# Supplementary material for: Reliability and validity of physical examination tests for the assessment of ankle instability
Source: Chiropr Man Therap. 2022 Dec 19;30:58. doi: 10.1186/s12998-022-00470-0 (PMC9764698; doi:10.1186/s12998-022-00470-0)
Supplement: Supplementary file 3 — Additional file 3. Orthopaedic tests for different types of ankle sprains. [file 12998_2022_470_MOESM3_ESM.pdf]

Additional File 3: Orthopaedic tests for different types of ankle sprains

Type of ankle injury	Potential ligaments affected	Orthopaedic tests that could be considered based on tests included in this study
**Syndesmosis injury**	• Anterior-inferior tibiofibular ligament<br>• Posteroinferior tibiofibular ligament<br>• Transverse tibiofibular ligament<br>• Interosseous membrane	• Tenderness of palpation of direct ligaments<br>• Squeeze test<br>• External rotation stress test<br>• Dorsiflexion compression test<br>• Cotton test<br>• Crossed-leg test
**Lateral ligament injury**	• Anterior talofibular ligament<br>• Posterior talofibular ligament<br>• Calcaneofibular ligaments	• Anterior drawer test<br>• Anterolateral drawer test<br>• Anterolateral talar palpation<br>• Reverse anterolateral drawer test<br>• Tenderness of palpation of direct ligaments<br>• Inversion stress test<br>• Talar tilt test
**Medial ligament (deltoid) injury**	• Anterior tibiotalar ligament<br>• Posterior tibiotalar ligament<br>• Tibionavicular ligament<br>• Tibiospring ligament<br>• Tibiocalcaneal ligaments	• Tenderness of palpation of direct ligaments<br>• Eversion stress test
