# Supplementary material for: Reliability and validity of physical examination tests for the assessment of ankle instability
Source: Chiropr Man Therap. 2022 Dec 19;30:58. doi: 10.1186/s12998-022-00470-0 (PMC9764698; doi:10.1186/s12998-022-00470-0)
Supplement: Supplementary file 4 — Additional file 4. Summary of the sensitivity and specificity values by orthopaedic test. [file 12998_2022_470_MOESM4_ESM.pdf]

Syndesmosis injury

External rotation test

Study	TP	FP	FN	TN	Sensitivity (95% CI)	Specificity (95% CI)
de Cesar 2011	2	7	8	39	0.20 [0.03, 0.56]	0.85 [0.71, 0.94]
Hosseinian 2021	6	2	21	76	0.22 [0.09, 0.42]	0.97 [0.91, 1.00]
Sman 2015	27	18	11	31	0.71 [0.54, 0.85]	0.63 [0.48, 0.77]

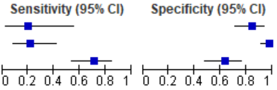

Squeeze test

Study	TP	FP	FN	TN	Sensitivity (95% CI)	Specificity (95% CI)
de Cesar 2011	3	3	7	43	0.30 [0.07, 0.65]	0.93 [0.82, 0.99]
Hosseinian 2021	7	1	20	77	0.26 [0.11, 0.46]	0.99 [0.93, 1.00]
Sman 2015	10	6	28	43	0.26 [0.13, 0.43]	0.88 [0.75, 0.95]

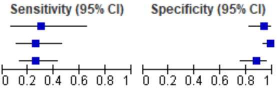

Dorsiflexion compression test

Study	TP	FP	FN	TN	Sensitivity (95% CI)	Specificity (95% CI)
Sman 2015	25	27	11	19	0.69 [0.52, 0.84]	0.41 [0.27, 0.57]

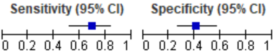

Tenderness syndesmosis

Study	TP	FP	FN	TN	Sensitivity (95% CI)	Specificity (95% CI)
Sman 2015	35	35	3	14	0.92 [0.79, 0.98]	0.29 [0.17, 0.43]
Van dijk 1996	53	4	69	34	0.43 [0.34, 0.53]	0.89 [0.75, 0.97]

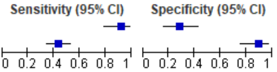

Lateral ligament injury

Anterior Drawer test

Study	TP	FP	FN	TN	Sensitivity (95% CI)	Specificity (95% CI)
Croy 2013	8	11	16	31	0.33 [0.16, 0.55]	0.74 [0.58, 0.86]
George 2020	13	0	9	13	0.59 [0.36, 0.79]	1.00 [0.75, 1.00]
Gomes 2018	7	0	7	10	0.50 [0.23, 0.77]	1.00 [0.69, 1.00]
Hosseinian 2021	77	2	18	8	0.81 [0.72, 0.88]	0.80 [0.44, 0.97]
Li 2020[a]	15	0	23	34	0.39 [0.24, 0.57]	1.00 [0.90, 1.00]
Li 2020[b]	2	0	36	34	0.05 [0.01, 0.18]	1.00 [0.90, 1.00]
Phisitkul 2009	6	1	2	1	0.75 [0.35, 0.97]	0.50 [0.01, 0.99]
Van dijk 1996	94	12	23	25	0.80 [0.72, 0.87]	0.68 [0.50, 0.82]
Vaseenon 2012	6	1	0	2	1.00 [0.54, 1.00]	0.67 [0.09, 0.99]

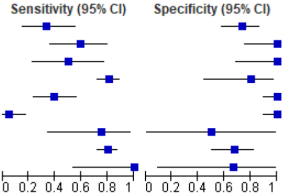

Anteriorlateral drawer test

Study	TP	FP	FN	TN	Sensitivity (95% CI)	Specificity (95% CI)
Li 2020[a]	19	1	19	33	0.50 [0.33, 0.67]	0.97 [0.85, 1.00]
Li 2020[b]	17	0	21	34	0.45 [0.29, 0.62]	1.00 [0.90, 1.00]
Phisitkul 2009	8	0	0	2	1.00 [0.63, 1.00]	1.00 [0.16, 1.00]
Vaseenon 2012	6	1	0	2	1.00 [0.54, 1.00]	0.67 [0.09, 0.99]

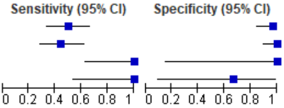

Tenderness of anterior talo-fibular ligament

Study	TP	FP	FN	TN	Sensitivity (95% CI)	Specificity (95% CI)
De Simoni 1996	30	0	0	0	1.00 [0.88, 1.00]	Not estimable
Van dijk 1996	122	26	0	12	1.00 [0.97, 1.00]	0.32 [0.18, 0.49]

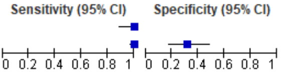

Tenderness of calcaneo-fibular ligament

Study	TP	FP	FN	TN	Sensitivity (95% CI)	Specificity (95% CI)
De Simoni 1996	17	3	8	2	0.68 [0.46, 0.85]	0.40 [0.05, 0.85]
Van dijk 1996	60	9	62	29	0.49 [0.40, 0.58]	0.76 [0.60, 0.89]

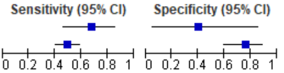

Reverse anterolateral drawer test

Study	TP	FP	FN	TN	Sensitivity (95% CI)	Specificity (95% CI)
Li 2020[a]	35	4	3	30	0.92 [0.79, 0.98]	0.88 [0.73, 0.97]
Li 2020[b]	33	3	5	31	0.87 [0.72, 0.96]	0.91 [0.76, 0.98]

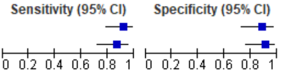

Talar tilt test

Study	TP	FP	FN	TN	Sensitivity (95% CI)	Specificity (95% CI)
George 2020	6	0	5	24	0.55 [0.23, 0.83]	1.00 [0.86, 1.00]
Rosen 2015	19	9	20	40	0.49 [0.32, 0.65]	0.82 [0.68, 0.91]

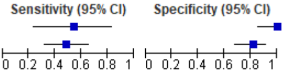

Inversion stress test

Study	TP	FP	FN	TN	Sensitivity (95% CI)	Specificity (95% CI)
Hosseinian 2021[c]	8	37	8	52	0.50 [0.25, 0.75]	0.58 [0.47, 0.69]
Hosseinian 2021[d]	42	3	42	18	0.50 [0.39, 0.61]	0.86 [0.64, 0.97]
Hosseinian 2021[e]	29	6	67	4	0.30 [0.21, 0.40]	0.40 [0.12, 0.74]

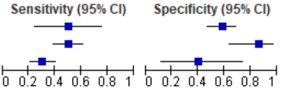

Anteriolateral talar palpation (modifed ADT)

Study	TP	FP	FN	TN	Sensitivity (95% CI)	Specificity (95% CI)
Gomes 2018	14	2	0	8	1.00 [0.77, 1.00]	0.80 [0.44, 0.97]

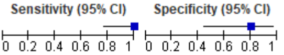

Medial ligament injury

Eversion stress test

Study	TP	FP	FN	TN	Sensitivity (95% CI)	Specificity (95% CI)
Hosseinian 2021	28	1	12	64	0.70 [0.53, 0.83]	0.98 [0.92, 1.00]

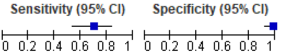

[a]Junior examiner; [b]Senior examiner, [c]posterior talofibular ligament, [d]calcaneofibular ligament, [e]anterior talofibular
